# Real-world outcomes of immunotherapy with or without chemotherapy in first-line treatment of advanced non-small cell lung cancer

**DOI:** 10.3389/fonc.2023.1182748

**Published:** 2023-06-19

**Authors:** Veronika Pelicon, Tanja Cufer, Lea Knez

**Affiliations:** ^1^ Department of Pharmacy, University Clinic Golnik, Golnik, Slovenia; ^2^ Medical Faculty, University of Ljubljana, Ljubljana, Slovenia; ^3^ Faculty of Pharmacy, University of Ljubljana, Ljubljana, Slovenia

**Keywords:** non-small cell lung cancer, immunotherapy, chemo-immunotherapy, realworld outcomes, first line

## Abstract

**Background:**

Immunotherapy alone (mono-IT) or combined with chemotherapy (chemo-IT) has recently become the cornerstone of first-line treatment for advanced non-small cell lung cancer (NSCLC) patients. Here, real-world outcomes of first-line mono-IT and chemo-IT of advanced NSCLC treated within routine clinical practice at a single academic center in the Central Eastern European (CEE) region are presented.

**Materials and methods:**

A total of 176 consecutive patients with advanced NSCLC treated with mono-IT (118 patients) or chemo-IT (58 patients) were included. At the participating institution, all medical data relevant for providing oncology care are collected prospectively and in a standardized manner using purposely created pro-forms. Adverse events (AEs) were recorded and graded according to Common Terminology Criteria for Adverse Events (CTCAE). The Kaplan−Meier method was used to estimate median overall survival (mOS) and median duration of treatment (mDOT).

**Results:**

The 118 patients in the mono-IT cohort had a median age of 64 years, most were male (59%), 20% had ECOG PS ≥2, and 14% had controlled CNS metastases at baseline. With a median follow-up time (mFU) of 24.1 months, the mOS was 19.4 months (95% CI, 11.1-27.6), and the mDOT was 5.0 months (95% CI, 3.5-6.5). The 1-year OS was 62%. The 58 patients in the chemo-IT cohort had a median age of 64 years, most were male (64%), 9% had ECOG PS ≥2, and 7% had controlled CNS metastases at baseline. With a mFU of 15.5 months, the mOS was 21.3 months (95% CI, 15.9-26.7), and the mDOT was 12.0 months (95% CI, 8.3-15.6). The 1-year OS was 75%. Adverse events of severe grade were recorded in 18% and 26% of patients, and immunotherapy discontinuation due to AEs occurred in 19% and 9% in the mono-IT and chemo-IT groups, respectively. No treatment-related deaths were recorded.

**Conclusion:**

The results from the present real-world observational study from a CEE country suggest similar effectiveness and safety of first-line mono-IT and chemo-IT in patients with advanced NSCLC to those observed in randomized clinical trials. However, continuous follow-up will offer better insight into the magnitude of long-term benefits in routine clinical practice.

## Introduction

Lung cancer is the leading cause of cancer-related death worldwide, with more than 2 million new cases and almost 1.8 million deaths per year. Non-small cell lung cancer (NSCLC) accounts for approximately 85% of all reported cases (1). Approximately half of these patients are diagnosed at an advanced stage, characterized by poor 5-year overall survival (OS) below 10% (2). For many decades, chemotherapy with platinum doublets was the only systemic treatment option for advanced NSCLC, achieving a median OS (mOS) of 8-14 months (1, 3). A trend towards lower mortality and higher survival rates was recently observed, especially for NSCLC patients (3). This improvement was mainly driven by introduction of novel treatment modalities in treatment of all stages of NSCLC. In general, discovery of oncogenic driver alterations and development of targeted therapies over the last two decades substantially contributed to these improvements. For instance, the sequential use of ALK-targeted agents resulted in mOS over 7 years in a real-world experience (3). However, these benefits are restricted to the minority of advanced NSCLC patients who harbor targetable driver alterations (1).

Only recently has treatment of advanced NSCLC patients without a targetable oncogene changed dramatically, owing to introduction of immunotherapy with immune check-point inhibitors (IT). Immunotherapy was first studied in the second-line setting, achieving substantial improvements in mOS over docetaxel chemotherapy (4–8). Even more impressive results were observed in the first-line setting, both with immunotherapy as monotherapy (mono-IT) and when combined with chemotherapy (chemo-IT) (9–17). In the pivotal KEYNOTE-024 trial, mono-IT with pembrolizumab led to mOS of up to 26.3 months, with a remarkable 5-year OS of 31.9%, in patients with programmed death ligand-1 (PD-L1) expression ≥50% (9). Moreover, a comparable mOS of up to 22.0 months was achieved in the PD-L1 unselected population when IT was combined with chemotherapy (12–17). Based on these results, immunotherapy with or without chemotherapy has become the cornerstone of first-line treatment of advanced NSCLC without targetable oncogenes. For patients with PD-L1 <50%, chemo-IT is the only approved and recommended treatment option; for patients with a PD-L1 ≥50%, both modalities, mono-IT and chemo-IT, are approved, with no randomized head-to-head comparison guiding treatment decisions (18). Based on high-quality observational data and pooled analysis of randomized clinical trials (RCTs), mono-IT is currently preferred for the vast majority of patients with PD-L1 ≥50% because it offers the advantage of avoiding chemotherapy toxicities without a significant impact on survival (19, 20).

However, translating evidence from RCTs to real-world circumstances can be challenging. Clinical trials are designed to maximize internal validity by enrolling patients with adequate organ function, good performance status, and no selected comorbidities. Consequently, a significant proportion of patients seen in daily practice are being excluded or underrepresented in clinical trials (21, 22). These differences result in a gap between the efficacy reported in RCTs and the effectiveness observed in routine clinical practice (21, 23). Thus, well-conducted real-world studies are strongly needed to inform about the effectiveness and safety of medical interventions outside clinical trial settings (21, 24). Real-world evidence is even more important in the case of completely new treatment modalities. Indeed, targeting the immune response with immune checkpoint inhibitors represents a specific approach to cancer treatment with inherited peculiarities and a unique set of immune-related adverse events (AEs). Moreover, in many countries with limited participation in clinical trials, including Slovenia, immunotherapy may have been first used in routine clinical practice, without previous expertise from clinical trials. These facts may have further hampered the outcomes of immunotherapy in routine clinical practice (21, 25).

As immunotherapy was first introduced in second-line treatment of advanced NSCLC, most published studies on immunotherapy real-world effectiveness are from this setting, and the findings are encouraging (26, 27). Real-world evidence on upfront mono-IT, albeit less robust, is also reassuring. Some of the largest series of advanced NSCLC patients with PD-L1 ≥50% report an encouraging mOS from 20 months to 26.5 months with first-line, mainly pembrolizumab, immunotherapy (28–34). Nevertheless, poorer mOS below 14 months was also reported in some large, multicentric observational trials (33, 35–40). Of note, in a large Dutch observational trial, which compared real-world outcomes with the results of the randomized trials, a significantly shorter OS was observed in a real-world setting (24). Even greater variability may be expected in real-world outcomes of immunotherapy when used in combination with chemotherapy, with its additional toxicities. Overall, there is a lack of evidence on the real-world effectiveness of chemo-IT, with only a few published studies reporting mOS between 13 months and 26 months (41–44). Moreover, the great majority of published real-world data on immunotherapy outcomes in NSCLC originate from North America, Western Europe and Japan and cannot be directly extrapolated to other health care settings (26, 33). Recently, our group reported some of the first real-world data for advanced NSCLC patients treated with immunotherapy in an academic center from the Central Eastern European (CEE) region, with survival and safety outcomes in the first-line and second-line setting largely comparable to those in clinical trials (45). However, the number of patients treated with first-line therapy was very limited, and no patient was treated with chemo-IT. In addition, to our knowledge, no real-world data on chemo-IT outcomes in NSCLC for CEE countries have been published. We therefore performed an observational study on the effectiveness and safety of first-line mono-IT and chemo-IT for patients with advanced NSCLC treated in everyday clinical practice at a single academic center in the Central Eastern European region.

## Materials and methods

### Study design and population

This observational cohort study included consecutive patients with pathologically confirmed advanced NSCLC treated with mono-IT or chemo-IT in the first-line setting in routine clinical practice at a single academic center in Slovenia between June 2017 and December 2021. All included patients tested negative for *epidermal growth factor receptor* (EGFR), *anaplastic lymphoma kinase* (ALK) and *ROS Proto-Oncogene 1* (ROS1) molecular alterations. *Kristen rat sarcoma oncogene* (KRAS) status and PD-L1 expression were evaluated in all included patients. All molecular testing was routinely performed according to the standard laboratory guidelines and quality control procedures valid at that time. PD-L1 testing was performed on formalin-fixed, paraffin-embedded histology samples or cytospins by using PD-L1 monoclonal antibodies (22C3 clone by DAKO, Glostrup, Denmark).

Patients were treated with pembrolizumab mono-IT, which was according to the registrational status restricted to patients with PD-L1 ≥50%, or chemo-IT combining atezolizumab or pembrolizumab with platinum-based chemotherapy, regardless of PD-L1 expression. Although chemo-IT was registered and reimbursed regardless of PD-L1 status, it was rarely used in patients with high PD-L1≥50% expression. The choice of treatment was at the oncologist’s discretion, but always based on international clinical practice guidelines valid at that time (46) and, in particular, addressing also drug availability. To be available, a drug had to have been granted marketing authorization by the European Medicines Agency (EMA) and have gained national reimbursement.

Patients were treated and followed up according to valid guidelines (46) within routine clinical practice and at a single institution. Patient functional status was assessed by the Eastern Cooperative Oncology Group (ECOG) Performance Status Scale (47). Clinicians were encouraged to record and grade AEs by Common Terminology Criteria for Adverse Events (CTCAE) valid at the time and to evaluate response to treatment according to RECIST 1.1 (48–50). Oncologists recorded all-cause AEs with no distinction of immune-related AEs (irAEs).

### Data collection

The study protocol was approved by the Medical Ethics Committee of the Republic of Slovenia (January 11, 2022; 0120-513/2021/3). All data were collected anonymously, and the need to obtain written informed consent from patients was waived due to the retrospective nature of the study. Data were obtained by reviewing the medical records of individual patients. At the participating institution, all medical data relevant for providing oncology care are collected prospectively and in a standardized manner using purposely created pro-forms. The date of censor for survival analyses was May 3, 2022.

### Study outcomes and statistical analysis

Results are presented separately for the mono-IT and chemo-IT cohorts. Patient and treatment characteristics were analyzed using descriptive statistics. The Kaplan−Meier method and 95% confidence interval (CI) were used to estimate medians for OS and duration of treatment (DOT) with immunotherapy. DOT was calculated as the time between the first and last dose of immunotherapy. OS was calculated from the start of treatment of interest until death from any cause. The median follow-up time was calculated using the reverse Kaplan−Meier method. Hazard ratios (HRs) and their 95% CIs were estimated using the Cox proportional hazards model in univariate analyses to evaluate the influence of patient baseline characteristics on OS. A p value of less than 0.05 was considered statistically significant. All statistical analyses were performed using SPSS version 28.0 (IBM Corporation).

### Results

Our observational study included a total of 176 patients with advanced NSCLC treated with mono-IT (118 patients) or chemo-IT (58 patients) in the first-line setting.

#### Mono-immunotherapy cohort

##### Patients and treatments

The 118 patients treated with mono-IT had a median age of 64 years (range, 39-81), 59% (70/118) of them were male, the majority (88%; 104/118) were former or current smokers, and 18% (21/118) had squamous histology (**Table 1**). The proportion of patients with PS ≥ 2 was 20% (23/118), and controlled CNS metastases were present in 14% (16/118) of the patients at baseline. All patients in the mono-IT cohort had PD-L1 expression ≥50%, with 47% (56/118) having very high PD-L1, i.e., ≥90%. KRAS-mutated NSCLC was present in 40% (47/118) of patients. All patients in the mono-IT cohort were treated with pembrolizumab.

**Table 1:** Baseline characteristics of patients treated with immunotherapy monotherapy (mono-IT) and in combination with chemotherapy (chemo-IT).

Characteristic		Mono-IT n=118 (100%)	Chemo-IT n=58 (100%)
Median age (range), years		64 (39–81)	64 (46-76)
Sex	Female	48 (41)	21 (36)
	Male	70 (59)	37 (64)
Smoking	Former or current	104 (88)	52 (90)
	Never	14 (12)	6 (10)
Histology	Squamous	21 (18)	15 (26)
	Nonsquamous	97 (82)	43 (74)
ECOG PS	0-1	95 (80)	53 (91)
	≥ 2	23 (20)	5 (9)
CNS metastasis	With	16 (14)	4 (7)
	Without	102 (86)	54 (93)
PD-L1	≥ 50%	118 (100)	5 (9)
	1-49%	0 (0)	31 (54)
	<1%	0 (0)	22 (38)
KRAS	Mutated	47 (40)	21 (36)
	Wildtype	71 (60)	37 (63)

CNS, central nervous system; ECOG, Eastern Cooperative Oncology Group; ICI. immune checkpoint inhibitor; IQR, interquartile range; KRAS, Kirsten rat sarcoma oncogene; PD-L1, programmed death-ligand 1; PS, performance status.

##### Effectiveness

**Figure 1:**
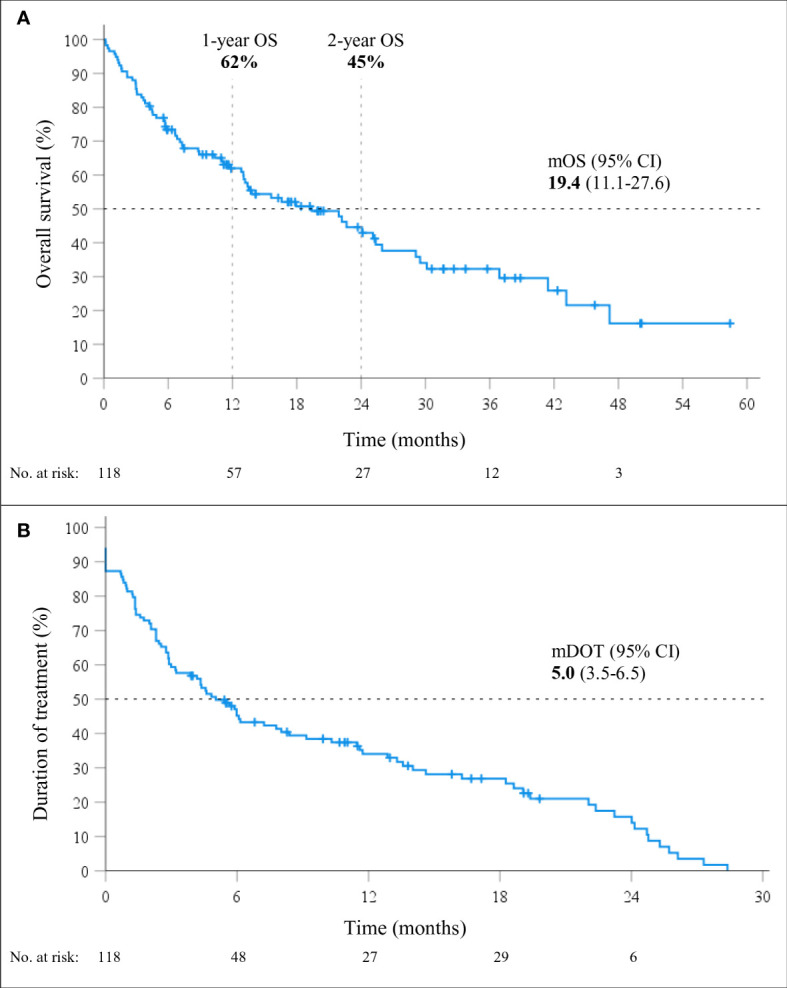
Overall survival (OS; **A**) and duration of treatment (DOT; **B**) in patients treated with immunotherapy monotherapy (mono-IT) (n=118).

At a median follow-up of 24.1 months (95% CI 13.2-25.1), mOS of 19.4 months (95% CI, 11.1-27.6; **Figure 1**) was observed. The 1-year and 2-year OS were 62% (95% CI, 53-71%) and 45% (95% CI, 34-55%), respectively. However, 26% (31/118) of patients died within six months from the start of immunotherapy and the baseline characteristics of these 31 patients are shown in **Table 2**. During the entire follow-up period, 58% (68/118) of the deaths occurred. The median DOT was 5.0 months (95% CI, 3.5-6.5). None of the baseline patient or tumor characteristics were associated with OS (**Table 3**).

**Table 2:** Baseline characteristics of the 31 patients treated with immunotherapy monotherapy (mono-IT) who died within six months from the start of immunotherapy.

Characteristic		Mono-IT n=31 (100%)
**Median age (range), years**		64 (39-81)
**Sex**	Female	15 (48)
	Male	16 (52)
**Smoking**	Former or current	27 (87)
	Never	4 (13)
**Histology**	Squamous	3 (10)
	Nonsquamous	28 (90)
**ECOG PS**	0-1	23 (74)
	≥ 2	8 (26)
**CNS metastasis**	With	6 (19)
	Without	25 (81)
**PD-L1**	≥ 50%	31 (100)
	1-49%	0 (0)
	<1%	0 (0)
**KRAS**	Mutated	16 (52)
	Wildtype	15 (48)

CNS, central nervous system; ECOG, Eastern Cooperative Oncology Group; ICI. immune checkpoint inhibitor; IQR, interquartile range; KRAS, Kirsten rat sarcoma oncogene; PD-L1, programmed death-ligand 1; PS, performance status.

**Table 3:** Univariate analyses of overall survival in patients treated with immunotherapy monotherapy (mono-IT) and in combination with chemotherapy (chemo-IT).

	Mono-IT		Chemo-IT		
	HR (95% CI)	P value	HR (95% CI)	P value	
For baseline characteristics					
Age of ≥65 years	1.29 (0.80-2.09)	0.299	1.73 (0.74-4.08)	0.205
Female sex	1.08 (0.67-1.76)	0.746	1.78 (0.78-4.03)	0.171
Non-smokers	1.16 (0.59-2.28)	0.660	1.84 (0.53-6.30)	0.331
Squamous	0.81 (0.42-1.54)	0.514	2.32 (0.98-5.47)	0.055
ECOG PS ≥2	1.58 (0.87-2.87)	0.135	3.72 (0.82-16.86)	0.088
CNS metastasis	1.06 (0.54-2.07)	0.876	0.39 (0.05-2.93)	0.357
PD-L1 <90% (mono-IT), <1% (chemo-IT)	1.49 (0.91-2.42)	0.112	1.66 (0.73-3.79)	0.226
KRAS mutated	0.89 (0.53-1.48)	0.647	0.76 (0.31-1.84)	0.540

CI, confidence interval; CNS, central nervous system; ECOG, Eastern Cooperative Oncology Group; HR, hazard ratio; IT, immunotherapy; KRAS, Kirsten rat sarcoma oncogene; PD-L1, programmed death-ligand 1; OS, overall survival; PS, performance status.

##### Safety

The incidence of all-cause AEs was 86% (102/118) among patients who received mono-IT (**Table 4**). Severe AEs, grade 3 or 4, were recorded in 18% (21/118) of patients. The most common AEs of any grade were fatigue, skin disorders, thyroid disorders, hepatotoxicity, diarrhea and arthralgia. A quarter (25%, 29/118) of the patients required treatment with systemic corticosteroids due to AEs and 19%.(23/118) required permanent treatment discontinuation due to an AE, which was grade 2 in 8 cases. The AEs that led to permanent discontinuation of immunotherapy were colitis (7 patients), hepatotoxicity (5 patients), pneumonitis (3 patients), skin disorders (2 patients), diarrhea (2 patients), and myocarditis, encephalitis, dyspnea and mesenteritis, each of them observed in one patient. Hospitalization due to AEs was required in 21% (25/118) of the patients. There were no treatment-related deaths.

**Table 4:** Adverse events in patients treated with immunotherapy monotherapy (mono-IT) and in combination with chemotherapy (chemo-IT).

	Mono-IT n=118 (100%)		Chemo-IT n=58 (100%)	
Adverse event	Any grade	Grade 3/4	Any grade	Grade 3/4
Any adverse event	102 (86)	21 (18)	55 (95)	15 (26)
Fatigue	67 (57)	0 (0)	45 (77)	0 (0)
Skin disorders	55 (47)	1 (1)	27 (46)	3 (5)
Hypo-/hyperthyroidism	24 (20)	2 (2)	17 (29)	0 (0)
Hepatotoxicity ^a^	23 (19)	5 (4)	21 (36)	3 (5)
Diarrhoea	17 (14)	6 (5)	12 (21)	0 (0)
Arthralgia	16 (14)	0 (0)	13 (22)	0 (0)
Colitis	10 (9)	4 (4)	2 (3)	0 (0)
Pneumonitis	9 (8)	2 (2)	3 (5)	2 (3)
Infection	7 (6)	1 (1)	8 (14)	3 (5)
Constipation	5 (4)	0 (0)	29 (50)	0 (0)
Infusion reactions	2 (2)	0 (0)	0 (0)	0 (0)
Anaemia	2 (2)	1 (1)	34 (59)	1 (2)
Stomatitis	2 (2)	0 (0)	14 (24)	0 (0)
Nausea	0 (0)	0 (0)	23 (40)	0 (0)
Vomiting	0 (0)	0 (0)	10 (17)	0 (0)
Neutropaenia	0 (0)	0 (0)	6 (10)	2 (3)
Thrombocytopaenia	0 (0)	0 (0)	6 (10)	0 (0)
Other	23 (20)	4 (4)^b^	15 (26)	2 (3)^c^
**Led to corticosteroid treatment**	29 (25)		8 (14)	
**Led to discontinuation of IT**	23 (19)		5 (9)	
**Led to hospitalisation**	25 (21)		13 (22)	
**Death**	0 (0)		0 (0)	

a Includes liver enzyme elevation and hepatitis.

b Other adverse events G3-4 in mono-IT: dyspnea (1), Cushing Syndrome and encephalitis (1), myocarditis (1), mesenteritis (1).

c Other adverse events G3-4 in chemo-IT group: nephritis (1), pancreatitis (1). IT, immunotherapy; G, grade.

#### Chemo-immunotherapy cohort

##### Patients and treatments

The 58 patients treated with chemo-IT had a median age of 64 years (range, 46-76), 64% (37/58) were male, the majority (90%; 52/58) were former or current smokers, and 26% (15/58) had squamous histology (**Table 1**). Only 9% (5/58) of these patients had PS ≥2. Controlled CNS metastases at baseline were present in 7% (4/58). PD-L1 was <1%, 1-49% and ≥50% in 38% (22/58), 54% (31/58) and 9% (5/58) of the patients, respectively. KRAS-mutated NSCLC was present in 36% (21/58) of the patients.

The great majority (56/58) of patients in the chemo-IT cohort received pembrolizumab with platinum-based chemotherapy. Overall, 69% (40/58) received pembrolizumab with platinum and pemetrexed and 28% (16/58) pembrolizumab with platinum and paclitaxel; 2 of 58 (3%) patients received atezolizumab, one with platinum and pemetrexed and one with platinum and gemcitabine.

##### Effectiveness

**Figure 2:**
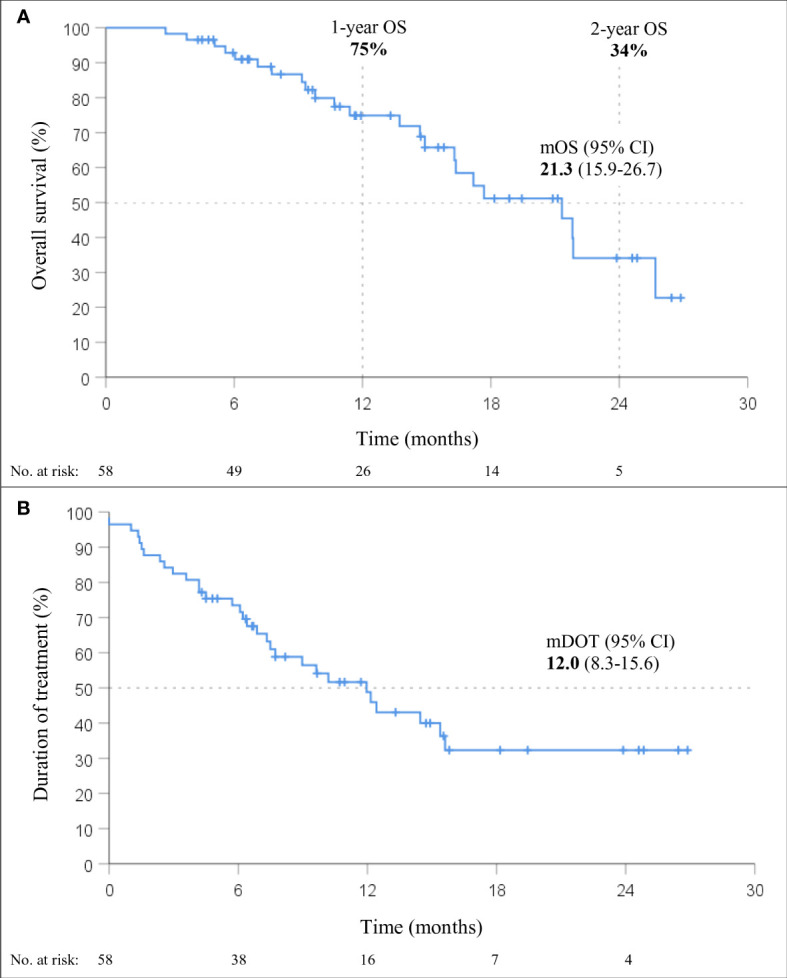
Overall survival (OS; **A**) and duration of treatment (DOT; **B**) in patients treated with immunotherapy in combination with chemotherapy (chemo-IT) (n=58).

At a median follow-up of 15.5 months (95% CI 11.0-20.1), mOS of 21.3 months (95% CI, 15.9-26.7; **Figure 2**) was observed. The 1-year and 2-year OS were 75% (95% CI, 62-87%) and 34% (95% CI, 15-54%), respectively. Within six months from the start of therapy, 7% (4/58) of the patients died; 40% (23/58) of the deaths occurred during the entire follow-up period. The median DOT with immunotherapy in the chemo-IT cohort was 12.0 months (95% CI, 8.3-15.6).

None of the baseline patient and tumor characteristics were associated with OS (**Table 3**). However, a trend towards shorter OS was observed in patients with squamous histology (p=0.055; HR 2.32, 95% CI: 0.98-5.47). In fact, the mOS of patients with nonsquamous and squamous histology was 21.8 months (95% CI, 17.5-26.2) and 16.3 months (95% CI, 12.9-19.7), respectively.

##### Safety

The incidence of all-cause AEs in the chemo-IT cohort was 95% (55/58, **Table 4**). Severe AEs, grades 3 or 4, were present in 26% (15/58) of the patients. The most common AEs of all grades were fatigue, anemia, constipation, skin disorders, and nausea; the most common severe AEs in this cohort of patients were skin disorders, hepatotoxicity, infection, neutropenia and pneumonitis. Treatment with systemic corticosteroids was required in 14% (8/58) of the patients, and permanent immunotherapy discontinuation due to AEs occurred in 9% (5/58). All AEs that led to immunotherapy discontinuation were grade 3. Of the 5 patients who discontinued immunotherapy in chemo-IT cohort 2 patients had pneumonitis, one experienced hepatotoxicity, one skin disorder and one neutropenia and arthralgia. Hospitalization due to AEs was required in 22% (13/58) of the patients. There were no treatment-related deaths.

## Discussion

The current study presents data on the effectiveness and safety of first-line mono-IT and chemo-IT in 176 patients with advanced NSCLC treated at a single academic center in a CEE country. The reported survival outcomes of mono-IT did not reach those of the pivotal KEYNOTE-024 study (9) but are in line with those reported in other RCTs and among the most favorable outcomes of published real-world studies (11, 28–33). Furthermore, the results for the chemo-IT cohort are similar to those published in RCTs (12–17) and within the large variability of the limited real-world data (41–44). Additionally, the safety outcomes of both mono-IT and chemo-IT (severe AEs in 18% and 26% of patients, respectively) did not differ substantially from the toxicity observed in pivotal trials (9, 10, 12–16, 51, 52).

Our cohort of 118 advanced NSCLC patients treated with first-line mono-IT within routine clinical practice achieved mOS of 19.4 months and 1-year OS of 62%, not reaching those of the pivotal KEYNOTE-024 clinical trial with mOS of 26.3 months and 1-year OS of 70% but similar to those reported for patients with PD-L1 expression ≥50% in other RCTs, with mOS of approximately 20 months (9–11). In fact, the reported outcomes are among the most encouraging within the large variability of other real-world studies, reporting mOS between 12.1 months and 26.5 months and 1-year OS between 53% and 60% (28–40, 53). The real-world evidence on upfront mono-IT in advanced NSCLC is indeed extensive, with reports including more than 500 patients (31, 32, 37, 53). Most importantly, in numerous real-world series, outcomes comparable to RCTs have been achieved despite inclusion of patients with less favorable prognosis, such as those with PS ≥2, who are excluded from RCTs, and a higher proportion of patients with CNS metastases than RCTs (29, 30, 32, 33). In our cohort of mono-IT patients, 20% had PS ≥2, and 14% had CNS metastases at baseline.

Despite the encouraging mOS of our patients treated with mono-IT, the initial steep decrease in the survival curve is alarming, with as many as 26% of the patients dying in the first six months from the start of treatment. These findings mimic those from RCTs, suggesting the existence of a subgroup of patients experiencing early disease progression and an early excess of death in the first months of treatment with mono-IT over chemotherapy (9–11). The baseline characteristics of our patients, dying within 6 months from the start of mono-IT largely resemble those of the entire mono-IT cohort. Early progression is indeed one of the major drawbacks of mono-IT and extensive research focuses on the search for predictive characteristics and biomarkers. The median duration of treatment with mono-IT of 5.0 months observed in our study is shorter than the median DOT of 7.9 months reported in KEYNOTE-024 (9). However, acknowledging that DOT with immunotherapy is not a good surrogate marker for OS, mDOT as such is of no concern. In fact, patients who discontinue immunotherapy early due to irAEs often experience long-term disease control with improved survival outcomes (54). What is worrisome is the high percentage of deaths observed during the first months of mono-IT, even in a cohort of patients with exclusively high PD-L1≥50%, expression.

Our cohort of 58 advanced NSCLC patients treated with chemo-IT in routine clinical practice achieved mOS of 21.3 months and 1-year OS of 75%. The mOS reported herein is similar with that of RCTs both in patients with non-squamous histology, being 21.8 months in our study compared to 18.6 to 22.0 months in RCTs (13, 14, 17), and in patients with squamous histology being 16.3 months in our study compared to 14.2 to 17.1 months in RCTs (12, 16). Moreover, similar mOS was achieved, even though a lower proportion (9%) of patients in our study than in RCTs had PD-L1 ≥50% (14-32%). Nevertheless, the median follow-up in our chemo-IT cohort was only 15.5 months and still much shorter than that in RCTs, with possible changes in outcomes as data mature (13–17, 55). As expected, real-world evidence on chemo-IT in first-line treatment of advanced NSCLC is still limited with regard to number and follow-up time because of the short time since regulatory approval and introduction of chemo-IT into everyday clinical practice. To date, real-world studies with chemo-IT show great variability in reported mOS, ranging from 12.7 to 25.6 months, with only a few studies reporting landmark 1-year OS due to short median follow-up (41–44). Surprisingly, the baseline characteristics of patients included in real-world studies of chemo-IT did not differ from those included in RCTs to the same extent as observed in patients treated with mono-IT. In fact, in our chemo-IT cohort, the proportion of patients with poor prognostic indicators was low. Only 9% of patients with PS ≥2 and 7% with CNS metastases were included, and the median age was 64 years. This is similar to RCTs and other real-world studies with no or only up to 12% of patients with PS ≥2, 7-18% of patients with CNS metastases, and patient median age of 63-66 years (13, 14, 16, 17, 41–43, 55). This judiciousness in selecting patients for chemo-IT treatment in routine clinical practice probably reflects concerns about chemotherapy toxicities in addition to immunotherapy treatment.

In addition to extra toxicities, combining chemotherapy with immunotherapy seems to lower the proportion of patients experiencing early disease progression, with only 7% of patients treated with chemo-IT dying in the first six months in our study compared to 26% in our mono-IT cohort. These findings mirror those of RCTs (12–17). In our cohort, the median DOT with chemo-IT was 12.0 months, longer than the 6.1-9.8 months in RCTs that reported this outcome (13–15, 17).

The real-world safety of immunotherapy treatment, either monotherapy or in combination with chemotherapy, is similar to that reported in the setting of clinical trials. In our mono-IT cohort, most patients (86%) experienced an AE, but it was severe, grade 3 or 4, in only 18% of patients. Interestingly, although the frequency of severe AEs was higher (more than 30%) in the pivotal trials, possibly due to underreporting in routine clinical practice, the discontinuation rate due to AEs in these pivotal trials was lower (14%) than in our cohort of patients (19%) (51, 56). In our study, even grade 2 AEs led to discontinuation of mono-IT in 8 patients; the limited experience with management of irAEs at the time of introduction of immunotherapy in first-line treatment may have dictated this cautionary approach. In our chemo-IT cohort, nearly all (95%) patients experienced an AE, which is in line with that (94% to 100%) reported in RCTs (13–17). Again, the rate of severe AEs in our study (26%) was substantially lower than the up to 81% reported in RCTs (14–16, 57, 58) However, discontinuation of immunotherapy due to AEs in our chemo-IT was lower (9%) than in RCTs (approximately 20%) (52, 58). Surprisingly, the 9% discontinuation rate was even lower than in our mono-IT cohort (19%), which might be due to the later introduction of chemo-IT into routine clinical practice and improved management of irAEs, as based on already existing international guidelines (59) and skills, obtained through treatment of patients with mono-IT. No new safety concerns and no treatment-related deaths were noted in our study of mono-IT and chemo-IT in advanced NSCLC.

In general, comparison of our safety outcomes with other real-world studies is difficult because only a minority of real-world studies report safety outcomes. In addition, studies report AEs differently, some report all AEs, whereas others report only irAEs or treatment-related AEs, leading to a very large variability in observed frequencies. The reported rates of AEs in mono-IT real-world studies range from the highest being very similar to those observed in our study (86%) and the lowest reporting any AE in only approximately one-third of patients (45). Chemo-IT real-world studies report AEs occurring in the majority of patients, with severe AEs observed in a wide range of patients, from 18% to 73% (60–62). Thus, although available real-world evidence seems reassuring, with similar or even lower rates of AEs than in RCTs, it should be acknowledged that the data are still very limited and that there is possible underreporting of AEs outside the clinical trial setting (45).

In our study, none of the baseline patient and tumor characteristics were associated with OS in either the mono-IT or the chemo-IT cohort. A trend towards shorter OS was observed only in patients with squamous histology treated with chemo-IT (p=0.055; HR 2.32, 95% CI: 0.98-5.47). This finding is not surprising, as in NSCLC, squamous histology is a known negative prognostic factor, and shorter OS was observed in patients with squamous histology treated with immunotherapy (63). More unexpected is the lack of association of well-established prognostic factors, such as poor PS or presence of CNS metastasis at baseline, possibly due to the small sample number of patients in each group. In fact, poor PS is repeatedly reported to be associated with poorer survival outcomes with immunotherapy. A recent meta-analysis estimated that OS rates were halved in NSCLC patients with poor PS ≥ 2 (33), and shorter OS was observed in real-world studies in patients with baseline CNS metastases (64). In our study, smoking status was not associated with survival outcomes, despite recent observations from RCTs and real-world studies uniformly showing worse performance of mono-IT in never-smokers than smokers (11, 65). Moreover, extremes in PD-L1 expression, PD-L1 ≥90% in the mono-IT cohort and PD-L1 <1% in the chemo-IT cohort, were not associated with better or worse OS, respectively, in our study. However, a meta-analysis of real-world studies showed that high PD-L1 expression (66) and very high PD-L1 ≥90% were associated with longer OS (67), highlighting the importance of interpreting PD-L1 status beyond the arbitrarily set threshold of 50% PD-L1 expression.

### Strengths and limitations

The key strength of our study is its in-depth reporting of adverse events. This was possible due to the precise monitoring and recording of AEs according to CTCAE criteria, as implemented in everyday clinical practice at our center years before this study. The weakness of our research is that it is a unicentric study with a relatively short observation period and a small number of included patients, particularly in the cohort treated with chemo-IT, which has been introduced into routine clinical practice only recently. Another weakness is that response to treatment was not strictly evaluated according to RECIST 1.1 criteria; therefore, overall response rates and median progression-free survival are purposely not reported here.

## Conclusion

The results from the present real-world observational study suggest similar effectiveness and safety of first-line mono-IT and chemo-IT in patients with advanced NSCLC to those observed in RCTs. This study contributes to the growing body of global evidence on the effectiveness and safety of mono-IT or chemo-IT as employed in everyday clinical practice. Describing our population and reporting outcomes might help both oncologists and patients in making decisions about this treatment. Importantly, the results of our research also provide health policy-makers with valuable data on the effectiveness of immunotherapy in routine clinical practice. This is of significance for health care systems in CEE, which are still struggling due to a lack of resources and a gap in cancer control compared to more developed countries in Western Europe. However, continuous follow-up of patients will offer further insight into the magnitude of long-term benefits in routine clinical practice.

## Data availability statement

The raw data supporting the conclusions of this article will be made available by the authors, without undue reservation.

## Ethics statement

The studies involving human participants were reviewed and approved by Medical Ethics Committee of the Republic of Slovenia (January 11, 2022; 0120-513/2021/3). Written informed consent for participation was not required for this study in accordance with the national legislation and the institutional requirements.

## Author contributions

All authors contributed to the study conception and design. VP and LK contributed to the data collection and statistical analysis. All authors interpreted the data and drafted the manuscript. All authors contributed to the article and approved the submitted version.
